# Reasons for non-vaccination in pediatric patients visiting tertiary care centers in a polio-prone country

**DOI:** 10.1186/0778-7367-71-19

**Published:** 2013-07-13

**Authors:** Asfandyar Sheikh, Bushra Iqbal, Anabia Ehtamam, Maria Rahim, Hiba Arshad Shaikh, Hina Azhar Usmani, Javeria Nasir, Sheharbano Ali, Muniba Zaki, Tooba Abdul Wahab, Warda Wasim, Ali Akber Aftab

**Affiliations:** 1 Dow Medical College, Dow University of Health Sciences, Baba-e-Urdu Road, Karachi 74200, Pakistan

## Abstract

**Background:**

The Expanded Program on Immunization (EPI) was initiated by World Health Organization (WHO) in 1974 in order to save children from life threatening, disabling vaccine-preventable diseases (VPDs). In Pakistan, this program was launched in 1978 with the main objectives of eradicating polio by 2012, eliminating measles and neonatal tetanus by 2015, and minimizing the incidence of other VPDs. However, despite the efforts of government and WHO, this program has not received the amount of success that was desired. Hence, the objectives of this study were to elucidate the main reasons behind not achieving the full immunization coverage in Pakistan, the awareness of children’s attendant about the importance of vaccination, their attitudes, thoughts and fears regarding childhood immunization, and the major hurdles faced in pursuit of getting their children vaccinated.

**Methods:**

This was an observational, cross-sectional, questionnaire-based study conducted during a one year period from 4th January, 2012 to 6th January, 2013 at the pediatric outpatient clinics of Civil Hospital (CHK) and National Institute of Child Health (NICH). We attempted to interview all the parents who could be approached during the period of the study. Thus, convenience sampling was employed. The parents were approached in the clinics and interviewed after seeking informed, written consent. Those patients who were not accompanied by either of their parents were excluded from the study. The study instrument comprised of three sections. The first section consisted was concerned with the demographics of the patient and the parents. The second section dealt with the reasons for complete vaccination or under-vaccination. The last section aimed to assess the knowledge, attitudes and beliefs of the respondents.

**Results:**

Out of 1044 patients, only 713(68.3%) were fully vaccinated, 239(22.9%) were partially vaccinated while 92(8.8%) had never been vaccinated. The vaccination status showed statistically significant association with ethnicity, income, residence, number of children and paternal occupation (p < 0.05 for all). The most common provocative factor for vaccination compliance was mass media (61.9%). The most common primary reason for non-vaccination was lack of knowledge (18.1%), whereas the most common secondary reason for non-vaccination was religious taboos (31.4%). Majority of the respondents demonstrated poor knowledge of EPI schedules or VPDs. However, most believed that there was a need for more active government/NGO involvement in this area.

**Conclusion:**

The most common primary reason for non-vaccination, i.e. lack of knowledge, and the most common secondary reason, i.e. religious taboos, imply that there is dire need to promote awareness among the masses in collaboration with NGOs, and major religious and social organizations.

## Background

The Expanded Program on Immunization (EPI) was initiated by World Health Organization (WHO) in 1974 in order to save children from life threatening, disabling childhood illnesses [1]. The aim was to immunize children against 6 vaccine-preventable diseases (VPDs) i.e. poliomyelitis, neonatal tetanus, measles, diphtheria, pertussis and tuberculosis. In Pakistan, this program was launched in 1978 with the main objectives of eradicating polio by 2012, eliminating measles and neonatal tetanus by 2015, and minimizing the incidence of other VPDs [2]. Later, vaccines against hepatitis B (2002), hemophilus influenzae type B (2008) and were also added with support from the government and development partners [3]. Table 1 provides an overview of the current EPI schedule in Pakistan.

**Table 1:** EPI schedule for vaccination in Pakistan

**Disease**	**Cause of infection**	**Vaccine**	**Doses**	**Age of administration**
Tuberculosis	Bacteria	BCG	1	Soon after Birth
Poliomyelitis	Virus	OPV	4	OPV0: Soon after birth
				OPV1: 6 weeks
				OPV2: 10 weeks
				OPV3: 14 weeks
Diphtheria	Bacteria	Pentavalent vaccine (DPT + HepB + Hib)	3	Penta1: 6 weeks
Tetanus	Bacteria			
Pertussis	Bacteria			Penta2: 10 weeks
Hepatitis B	Virus			
Haemophilus Influenzae	Bacteria			Penta3: 14 weeks
Streptococcus Pneumoniae	Bacteria	Pneumococcal conjugate vaccine (PCV10)	3	Pneumo1: 6 weeks
				Pneumo2: 10 weeks
				Pneumo3: 14 weeks
Measles	Virus	Measles vaccine	2	Measles1: 9 months
				Measles2: 15 months

According to the 2010 report of Pakistan Institute of Legislative Development and Transparency (PILDAT), presently 15% of deaths below the age 5 years make up 50% of overall mortality in Pakistan, showing children as the most neglected part of Pakistan’s society [4]. Although there has been a big improvement in EPI coverage in Pakistan in recent years, stronger measures are still required to achieve the desired results. The percentage of Pakistani children (age 12–23 months) receiving all the vaccines is only 47%, and 6% of children remain non-vaccinated [4]. Complete EPI coverage from province to province also shows marked variation, being 35% in Baluchistan, 37% in Sindh, 53% in Punjab, 47% in Khyber Pakhtunkhwa (KPK) [4]. Routine EPI coverage as of 2007 was: BCG - 89%, Polio - 83%, DPT - 83%, Hepatitis B - 83% and Tetanus Toxoid - 46% [5]. These numbers showed improvements in 2012 to 95%, 89%, 89%, 89% and 68% respectively [5]. Despite the mentioned coverage, Pakistan is still among the 3 countries of the world where wild poliovirus remains endemic.

The government of Pakistan provides only 20% of total immunization expenditure while, EPI with the help of UNICEF, WHO, GAVI-ALLIANCE shares the major burden of funding [6]. Despite the funding provided by EPI and routine immunization coverage, Pakistan has seen outbreaks of measles and diphtheria in 2012, and an increased in number of tuberculosis and tetanus cases due to weak implementation and the major focus of government being on the polio eradication [7].

About 1000 deaths of children less than 5 years of age will occur if the EPI program is discontinued, which signifies its importance [8]. However, despite the efforts of government and WHO, this program has not received the amount of success that was desired. This has been due to a multitude of reasons, majority of which can be broadly divided into two main categories, namely provider-associated and consumer-associated. The former, which includes entities such as lack of proper government policies, absenteeism of vaccination personnel, poor vaccine quality and inadequate coverage, has been widely covered in local as well as international media [9-12]. In contrast, the latter has rarely received the attention it requires, which is evident in the paucity of pertinent cross-sectional literature from this geographical area. Included in this category are factors such as lack of awareness, illiteracy, social/religious dilemmas and misconceptions, financial issues and accessibility problems.

Various studies have been conducted in different parts of the world to derive the major factors associated with non-vaccination. For example, in a study conducted in Turkey, the most common reason for non-vaccination was lack of awareness, followed by non-compliance of spouse, health status of the child and missed opportunities [13]. On the other hand, a study from Philippines supported maternal illiteracy as the major cause of under-vaccination [14]. Studies from other parts of the world have reported similar reasons [15-17]. A systematic review taking into consideration all articles published between 1999 and 2009 was conducted by Rainey et al. [18]. The review divided the reasons for under-vaccination into four categories, i.e. those related to immunization systems, communication and information, family characteristics and parent attitudes or knowledge [18].

### Rationale and objectives

As mentioned earlier, there is not much data available from Pakistan regarding the failure of EPI in eradicating these preventable childhood diseases, especially from a tertiary care setting. Whatever is available is severely lacking in quality and focuses on the percentage of vaccination coverage, without fully covering the basic reasons coming in the way of achieving a safe childhood. Therefore, the aim of our research was to elucidate the main reasons behind not achieving the full immunization coverage in Pakistan, the awareness of children’s attendant about the importance of vaccination, their attitudes, thoughts and fears regarding childhood immunization, and the major hurdles faced in pursuit of getting their children vaccinated.

## Methods

### Study setting

This was an observational, cross-sectional, questionnaire-based study conducted during a one year period from 4th January, 2012 to 6th January, 2013 at the pediatric outpatient clinics of Civil Hospital (CHK) and National Institute of Child Health (NICH), which are two of the largest public sector, tertiary care hospitals in Metropolitan Karachi. These hospitals provide subsidized healthcare to patients, majority of whom belong to low socio-economic class.

### Study participants

In view of our research objectives, we attempted to interview all the parents who could be approached during the period of the study, and whose accompanying child was at least two years of age, but not more than 15 years. Thus, convenience sampling was employed. Those patients who were not accompanied by either of their parents were excluded from the study in order to avoid erroneous reporting. Those who had congenital malformations were also excluded, as were those whose parents gave consent in the negative.

### Study protocol

The parents were approached in the clinics and interviewed after seeking informed, written consent. The vaccination status of the accompanying child was ascertained from the appropriate documentation. In case a document was not available, the parents were inquired about the status. For those who were oblivious of the vaccine names, an approach based on vaccination schedules was adopted. For example: “Was your child vaccinated at the age of 1.5 months? If yes, then how many vaccines did he receive?” (An answer of 2 denotes completion of OPV1 and Penta1).

### Ethical review

The Ethical Review Board of Dow University of Health Sciences approved the study. The respondents were informed of their right to refuse at any time of the study. Confidentiality and anonymity of the data was maintained at all times. The protocol was designed according to the guidelines laid down by the Helsinki Declaration [19].

### Operational definitions

#### ***Fully vaccinated***

Children who had received the full course of vaccinations according to their age as per EPI schedule (see Table 1). Note that the pneumococcal vaccine has only been introduced recently, and was not investigated in our study.

#### ***Under-vaccinated***

Children who had either not been vaccinated (non-vaccinated) or had failed to complete the course of vaccinations (partially vaccinated) according to their age as per EPI schedule (see Table 1).

#### ***Partially vaccinated***

Children who had been vaccinated at least once, but had failed to complete the course of vaccinations according to their age as per EPI schedule (see Table 1).

#### ***Non-vaccinated***

Children who had never been vaccinated.

### Study questionnaire

The study instrument was designed with the help of the Departments of Pediatrics and Community Medicine, Dow University of Health Sciences. A group of parents was initially approached and presented with a number of open-ended questions. The output was then incorporated with a thorough review of the literature in order to design the best possible questionnaire. A pretest of this preliminary questionnaire was done on a sample of 25 parents and the questionnaire was edited accordingly.

The study instrument comprised of three sections. The first section consisted of two parts. Section 1A was concerned with the bio-data of the current child and included variables such as age, gender and a history of current or previous VPDs. Section 1B was concerned with the bio-data of the parents, and inquired about their educational status, occupation and financial status. The second section again comprised of two sections. Questions in Section 2A were only asked from the parents of fully vaccinated patients, and included reasons that convinced them to get their child vaccinated. On the other hand, questions in Section 2B were only asked from the parents of under-vaccinated patients, and included reasons that prevented them from getting their child vaccinated. These were divided into “primary” and “secondary”. The primary reason referred to the single most important reason reported by the respondents for non-vaccination, when asked “Why did you not get your child vaccinated?” The question had an open-ended connotation, and no options were given in this case. The response was then classified according to a preformed list of reasons. After the respondent had successfully answered the question, the reasons (excluding the primary reason) were inquired individually and the respondent was supposed to answer using a Yes/No approach, meaning that the respondents were allowed to pick more than one reason, as opposed to primary reason, where they were allowed to pick only one. The last section aimed to assess the knowledge of the respondents.

At the end of each interview, the parents were counseled on the need and positive aspects of vaccination, and attempts were made to shun any false beliefs. Hence, our study not only provided exploratory analysis, but also served as a didactic tool.

### Analysis of data

Data from the questionnaire was entered in SPSS (Statistical Package for the Social Sciences) version 17 for analysis and the results were compared. Descriptive statistics formed the mainstay of the statistical analysis. P values were calculated to determine the significance of association between variables and were based on the Chi-square test. Continuous variables such as age were converted into categorical ones. A P value of less than 0.05 was considered to be significant. Characteristics that were found to be significantly associated with vaccination status were entered into a multivariate logistic regression model. Vaccination status was converted into a dichotomous variable (vaccinated vs. under-vaccinated), and was used as the dependent variable for the regression model.

## Results

### Demographics

A total of 1044 out of 1200 parents approached agreed to the interview, giving a response rate of 87.0%. The mean age of the patients was 4.8 ± 2.9 years. 553(53.0%) patients were female, whereas 491(47.0%) were male. 969(92.8%) had both parents who were alive, whereas 34(3.3%) had only the mother alive, and 41(3.9%) had only the father alive. Measles (9.2%) was the most common previously contracted VPD, followed by Hepatitis B (6.4%), pertussis (3.6%), tuberculosis (1.8%) and polio (0.3%).

The mean maternal and paternal ages were 28.7 ± 4.1 years and 33.4 ± 5.9 years respectively. The mean number of years since marriage was 9.8 ± 4.7 years, with each couple having an average of ~3 children. The socioeconomic status of majority was low, with the mean household income being Rs. 7823.5 ± 2941.8 (~USD 80 ± 30). Table 2 gives a summary of the parent demographics.

**Table 2:** Demographics of parents at two tertiary care centers in Karachi, Pakistan from 4th January, 2012 to 6th January, 2013 and the association with the vaccination status

		**N**	**%**	P-value*
Ethnicity	Sindhi	293	28.1	**<0.001**
	Punjabi	109	10.4	
	Balochi	53	5.1	
	Pathan	187	17.9	
	Muhajir	341	32.7	
	Other	61	5.8	
Religion	Islam	939	89.9	0.103
	Christianity	27	2.6	
	Hinduism	73	7.0	
	Sikhism	4	0.4	
	Other	1	0.1	
Residence	Karachi	812	77.8	**0.002**
	Other City	71	6.8	
	Village	161	15.4	
Maternal Education	None	974	93.3	0.078
	Prim/Sec	49	4.7	
	Matriculation	20	1.9	
	Graduate	1	0.1	
Paternal Education	None	923	88.4	0.061
	Prim/Sec	78	7.5	
	Matriculation	39	3.7	
	Graduate	4	0.4	
Maternal Occupation	None	842	80.7	0.075
	Unskilled	173	16.6	
	Skilled	27	2.6	
	Business	2	0.2	
Paternal Occupation	None	347	33.2	**0.045**
	Unskilled	468	44.8	
	Skilled	151	14.5	
	Business	78	7.5	
Own House	Yes	401	38.4	0.112
	No	643	61.6	
Own Transport	Yes	253	24.2	0.095
	No	791	75.8	

*P-value based on Chi-square testing for the association between demographic factors and vaccination status.

### Vaccination status

**Figure 1:**
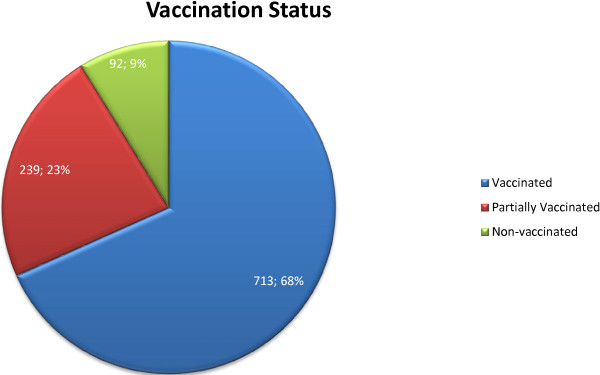
Vaccination status of patients at two tertiary care centers in Karachi, Pakistan from 4th January, 2012 to 6th January, 2013.

Out of 1044 patients, only 713(68.3%) were fully vaccinated, 239(22.9%) were partially vaccinated while 92(8.8%) had never been vaccinated. Figure 1 gives a graphical representation of this data. Out of those who were partially vaccinated, 107(44.8%) had not received the full course of Oral Polio Vaccine, and 51(21.3%) did not have a BCG scar. The vaccination status showed statistically significant association with ethnicity, income, residence, number of children and paternal occupation (p < 0.05 for all). However, no significant association was found with maternal or paternal education status (p > 0.05 for both). The significant variables were subjected to multivariate logistic regression analysis. The results are presented in Table 3.

**Table 3:** Multivariate logistic regression model to identify risk factors for under-vaccination at two tertiary care centers in Karachi, Pakistan from 4th January, 2012 to 6th January, 2013

		**aOR (95% CI)**
Ethnicity	Punjabi	1
	Sindhi	1.92 (1.06-3.19)
	Balochi	2.41 (1.14-5.46)
	Pathan	6.73 (2.23-14.66)
	Muhajir	1.07 (0.45-2.71)
Income	>Rs. 7,500	1
	Rs. 0 – Rs. 7,500	3.87 (1.27-12.14)
Residence	Karachi	1
	Other City	1.69 (1.01-2.93)
	Village	5.38 (2.14-11.39)
Number of Children	1-3	1
	>3	2.55 (1.24-5.32)
Paternal Occupation	Employed	1
	Unemployed	4.11 (1.31-12.23)

### Reasons for vaccination

The most common provocative factor for vaccination compliance was mass media (61.9%), followed by relative/friend (41.2%), family physician (39.7%), lady health worker (31.6%), spouse (11.4%) and NGO (8.8%). 131(18.4%) respondents reported that seeing other children with VPDs convinced them to get their children vaccinated.

### Reasons for non-vaccination

The most common primary reason for non-vaccination was lack of knowledge (18.1%), followed by lack of time due to busy schedule (12.4%), non-compliant spouse (10.6%), security conditions (8.4%), religious taboos (8.2%), lack of trust on medical facilities (6.3%), missed opportunities (5.7%), fear of side effects (5.1%), accessibility problems (4.8%), financial problems (4.2%), vaccination not deemed necessary (3.9%), physician advising against vaccination (3.6%), vaccination not considered effective (3.3%), poor previous experience with physicians (2.4%), VPDs not considered severe (1.5%), fear of exposing child to needles (0.9%) and being single parent (0.6%).

**Figure 2:**
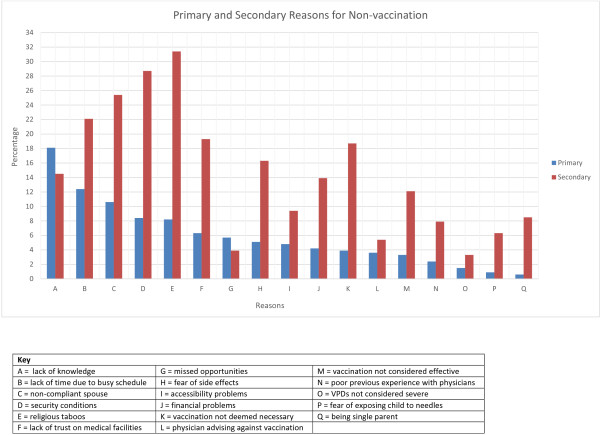
Primary and secondary reasons for non-vaccination at two tertiary care centers in Karachi, Pakistan from 4th January, 2012 to 6th January, 2013.

The most common secondary reason for non-vaccination was religious taboos (31.4%), followed by security conditions (28.7%), non-compliant spouse (25.4%), lack of time due to busy schedule (22.1%), lack of trust on medical facilities (19.3%), vaccination not deemed necessary (18.7%), fear of side effects (16.3%), lack of knowledge (14.5%), financial problems (13.9%), vaccination not considered effective (12.1%), accessibility problems (9.4%), being single parent (8.5%), poor previous experience with physicians (7.9%), fear of exposing child to needles (6.3%), physician advising against vaccination (5.4%), missed opportunities(3.9%) and VPDs not considered severe (3.3%). Figure 2 gives a graphical comparison of the primary and secondary reasons.

### Knowledge, beliefs and attitudes

When asked to name the disease which had been eradicated from the world due to successful vaccination campaigns, only 0.8% were able to name small pox. However, more people (1.2%) were successful in naming polio as the disease that had been eradicated from all countries except three. When asked to rate the importance of vaccination on a Likert scale of 1–5 (with 5 denoting very important and 1 denoting no importance), 52.9% considered it to be very important (mean 4.5 ± 0.5).

Only 2.8% were able to fully recall EPI vaccination schedule, whereas 12.4% were only partly successful. The most common perceived adverse reaction of vaccination was pain (61.4%), followed by fever (17.5%), swelling (6.5%), vomiting (3.4%) and diarrhea (1.1%). When asked to rate the severity of VPDs on a Likert scale of 1–5 (with 5 denoting very severe and 1 denoting not severe), 47.1% considered them to be very severe (mean 4.2 ± 0.9).

When asked to rate the importance of maternal vaccination during pregnancy on a Likert scale of 1–5 (with 5 denoting very important and 1 denoting no importance), 58.8% considered it to be very important (mean 4.4 ± 0.9). 79.6% reported that they would rather have the child vaccinated at home than visit a center. 71.9% said that they would advise others to get their children vaccinated. 87.7% were In favor of more active government/NGO involvement.

## Discussion

Although a number of studies aimed at assessing the vaccination status have been published, this is the first article from the Eastern Mediterranean Region that attempts to elucidate the reasons behind non-vaccination, with particular emphasis on the knowledge, attitudes and beliefs of the respondents [13,20-22]. Tertiary care centers were chosen as the study setting in order to determine the main factors hampering vaccination compliance in those who are successful in visiting these centers at least once in their lifetime.

The rate of complete vaccination coverage in our sample was 68.4%. This is similar to a study conducted in Nigeria, better than that in Ethiopia, but lower than those conducted in Turkey [13,20-22]. It was better than previous studies conducted in Pakistan (44.8% and 48%) [23,24]. However, since our sample represents patients visiting tertiary care centers, it lacks comparability with other studies. Moreover, the rate of vaccination in our sample is still too low, given the fact that most tertiary care centers in Pakistan have dedicated immunization clinics. In our study, the Pathan ethnicity had higher odds of being under-vaccinated. It is worthwhile to note here that illegal migration from Afghanistan to Pakistan is still rampant even after 23 years of the Afghan War. Most Afghan migrants report themselves as belonging to the Pathan ethnicity in order to avoid immigration laws. These migrants are usually under-vaccinated and may be responsible for transmitting vaccine preventable diseases such as polio across the border.

Although mass media campaigns remained the most common sources of vaccination-related information, the most common primary reason for non-vaccination was still lack of knowledge. This domain includes, but is not limited to, illiteracy, lack of awareness and misconceptions. This points towards an inherent defect in the advertisement campaigns. Most of these campaigns utilize channels such as televisions or newspapers, which fail to fulfill their purpose for the large proportion of illiterate population that lives in urban and rural areas. Although National Immunization Days are observed each year, and Pakistan has a large force of lady health workers, most of the areas still remain inaccessible owing to physical and political hurdles. The socioeconomic status (SES) for majority is low, and this factor has been shown to independently affect the rates of vaccination in previous studies [25]. In the study conducted by Williams et al., low SES children in urban areas were more prone to be under-vaccinated compared to high SES in rural areas [26]. Furthermore, failure to vaccinate simultaneously had a higher effect on predicting vaccination coverage for those having low SES [26]. The head of the family, in most cases, is usually a male, who occupies a dominant role in the Pakistani society. Hence, a non-compliant spouse is an important obstacle in successful completion of vaccination, as indicated by the fact that it was the third most commonly reported primary and secondary reason in our study.

Religious taboos carry special significance with regards to the under-vaccinated population of this area. The majority of the population is Muslim, and although Islamic literary sources (Quran and Hadith) encourage any efforts made for the improvement of health, most religious leaders harbor a disagreement against vaccination and seem to fulfill their own personal agenda. During our study, we encountered statements such as:

• “The *imam* (religious leader) has forbidden the use of vaccines as they contain porcine components.”

• “Vaccination is a conspiracy of the Zionists. Vaccinating our children, will inevitably make them sterile.”

This notion implies that steps should be taken religious organizations should be included in all future strategies, as they exercise a significant amount of influence over the Pakistani population.

Karachi remains the only metropolitan city where wild poliovirus is still endemic. Although the government has taken steps under the Polio eradication initiative, the number of polio cases have shown no signs of decline. The law and order situation of the city has a special role in exacerbating this problem, which is reflected by “security conditions” being the fourth most common primary reason and the second most common secondary reason in our study. The recent wave of target killings and terrorist attacks have stricken fear inside parents and has forced them not to bring their children for routine immunizations. Moreover, recent attacks have particularly targeted vaccination personnel, which has further dented the way to EPI success [27].

The strength of our study lies in the large number of parents interviewed. Previous studies have targeted communities for the extraction of data. We chose a tertiary care hospital in order to provide a different perspective with regard to our study topic. Future studies may improve upon our results by comparing reasons for non-vaccination in a community with those encountered at a tertiary care center. All attempts were made to ensure that the data collected was reliable and the methods were reproducible. However, our study was not free from limitations. The most important limitation for our study was that it was conducted at just two institutes. Although, these hospitals consist of a heterogeneous population coming from different backgrounds, they cannot be used to predict the overall situation in the country. Furthermore, convenient sampling was employed, which may have led to selection bias, and hence is not truly representative of the population under study. However, since this was just an observational study, the sampling method did seem to fulfill its purpose. Another limitation that could have affected the outcome of our study is the possibility of recall bias with regard to vaccination status. However, since our main aim was to elucidate the reasons for non-vaccination, recall bias may not have played a significant role.

## Conclusion

The most common primary reason for non-vaccination, i.e. lack of knowledge, indicates that the ongoing advertisement campaigns have partially failed to achieve the success desired, whereas the most common secondary reason, i.e. religious taboos, implies that many people believe that vaccination is forbidden in religion, a misconception that is further propagated by religious leaders. Hence, there is dire need to promote awareness among the masses in collaboration with NGOs, and major religious and social organizations.

## Competing interests

The authors declare that they have no conflicts of interests.

## Authors’ contributions

AS conceived the topic of the study and was involved in designing the study and analyzing data. BI, AE, MR, HS, HA, JN, SA, MZ, TW, WW and AA were involved in data collection. AS was involved in drafting the initial manuscript. BI, AE, MR, HS, HA, JN, SA, MZ, TW, WW and AA critically revised the manuscript, and their names are listed in decreasing order of their contributions. All authors have read and approved the final manuscript.
